# P064 When mind matters: a complex combination of giant cell arteritis, steroids and psychiatric history

**DOI:** 10.1093/rap/rkag113.094

**Published:** 2026-11-03

**Authors:** Harini Samarasinghe, Sophia Kostich, Emma Dolphin, Jessica Gunn

**Affiliations:** Cheltenham General Hospital, Cheltenham, United Kingdom; Cheltenham General Hospital, Cheltenham, United Kingdom; Cheltenham General Hospital, Cheltenham, United Kingdom; Cheltenham General Hospital, Cheltenham, United Kingdom

## Abstract

**Introduction:**

Giant cell arteritis (GCA) is a rheumatological emergency requiring prompt recognition and treatment to minimise irreversible complications such as permanent visual loss. Patients typically present via primary care to acute medical teams or directly to ophthalmology with visual symptoms. We describe a patient presenting with evolving visual disturbance whose management was complicated by multimorbidity and a complex past medical history, limiting the use of standard high-dose corticosteroid therapy requiring deviation from the normal treatment pathway. This case highlights the need for an individualised approach with regular multidisciplinary (MDT) input and demonstrates that straightforward conditions may not follow conventional treatment algorithms.

**Case description:**

A 76-year-old woman with a history of polymyalgia rheumatica, atrial fibrillation, severe major depression and psychosis, requiring sectioning under the Mental Health Act, presented to ophthalmology with sudden vision loss in her right eye. She had experienced increasing fatigue and declining mobility over several weeks. Ophthalmological examination demonstrated right optic disc swelling with a relative afferent pupillary defect. GCA was suspected; oral prednisolone 60mg daily, urgent temporal artery ultrasound (TAUSS) and rheumatology referral were arranged.

Blood tests demonstrated a C-reactive protein (CRP) of 13mg/L. TAUSS showed bilateral hypoechoic halo signs with positive compression signs, together with occlusion of the frontal and parietal branches, confirming GCA. Within days, she developed new visual symptoms affecting the left eye with optic disc swelling, requiring admission for intravenous methylprednisolone.

Given her psychiatric history and an incidental vertebral fracture, early advice was sought from the local tertiary rheumatology team who supported the use of tocilizumab as a steroid-sparing agent. She received two doses of intravenous tocilizumab 4-weeks apart, before switching to long-term subcutaneous (SC) treatment whilst steroids were tapered. A few weeks into steroids, she developed increasing agitation and medication refusal, requiring covert administration under the Mental Capacity Act (MCA). A cautious but more rapid steroid taper was adopted with close monitoring.

Admission exceeded two months, requiring regular psychiatry input. It was complicated by worsening mobility, which prompted CT brain imaging, demonstrating bilateral cerebellar infarcts. This necessitated further therapies input, complicating both treatment and discharge planning. Despite physical and psychiatric decline, she responded well to treatment with intravenous tocilizumab, and her clinical symptoms stabilised and CRP normalised. Discharge planning required careful consideration of suitable administrative methods given her fluctuating mental state and limited social support. Social circumstances created challenges in arranging ongoing treatment. To date, she remains in-patient with uncertain discharge outcomes.

**Discussion:**

This case outlines a GCA presentation where clinical decisions were shaped by evolving visual symptoms, multimorbidity, psychiatric history and frailty. Steroids were escalated promptly from oral prednisolone to intravenous methylprednisolone when contralateral visual symptoms developed to prevent further ischaemic optic nerve damage. Behavioural disturbance, worsened by admission and high-dose steroids, necessitated covert administration (under MCA) and early steroid-sparing therapy. Prompt escalation to intravenous tocilizumab aimed to reduce steroid-related psychiatric harm and mitigate bone fragility in the context of frailty. Once remission was established, a cautious but rapid steroid taper was initiated alongside tocilizumab.

GCA predominantly affects older adults, making steroid-related risks an inherent challenge. BSR guidance recommends assessing glucocorticoid-relevant comorbidities at diagnosis. Although GCA is common, standard management here was constrained by multimorbidity and severe psychiatric vulnerability. The case progression was atypical; visual symptoms progressed rapidly despite high-dose oral prednisolone and initial CRP was low (13mg/dL) despite ultrasound and ophthalmological confirmation.

While tocilizumab is typically used in relapsing disease, it is underutilised as an early steroid-sparing agent. Tocilizumab was favoured over methotrexate due to its more rapid onset, stronger evidence base and parenteral route, which was advantageous given covert medication needs and adherence concerns. Early MDT involvement was crucial to guide commencement of tocilizumab and the rapid steroid taper, emphasising the need for individualised management. Given the complexity, early pharmacy and social planning were essential.

Several questions arise from this case. While SC delivery is the standard outpatient regimen, the patients fluctuating mental state and limited social support challenged discharge planning, prompting consideration of community support and ongoing intravenous therapy. Furthermore, monitoring on tocilizumab is challenging due to CRP suppression, requiring heavier reliance on clinical symptoms, and whilst this remained easier to assess as an inpatient, we are still yet to see the practicality of this as an outpatient.

**Key learning points:**

• Two key learning points arise from this case. Firstly, individualised management is essential in elderly, multimorbid patients with GCA, aligning with BSR guidance on baseline steroid assessments. Standard pathways must remain flexible, and early MDT input is vital when evidence-based guidance is limited. Secondly, effective long-term management requires planning beyond the acute phase. IL-6 inhibition suppresses traditional inflammatory markers, necessitating a precise clinical monitoring plan. Furthermore for complex patients, clear communication of the follow up, transport and appointment scheduling, is essential. Ongoing biologic delivery and administration must be carefully coordinated with relevant teams in those who are vulnerable or socially unsupported, with a clear plan established prior to discharge.

• Additional discussion relates to optimising existing GCA pathways for high-risk cohorts. Given the recommended steroid prescribing assessment, a formal psychiatric risk assessment should be incorporated into GCA care pathways, particularly for patients with relevant histories to reduce the risk of steroid-induced psychiatric issues in vulnerable patients. Crucially, explicit discussion of mental health side effects must form a routine part of clinical care and drug counselling, so patients and families are fully informed.

• Secondly, prolonged hospitalisation exposes elderly patients to high risk of hospital acquired infections, deconditioning and cognitive decline, particularly alongside psychiatric comorbidities. Early, proactive discharge planning should be prioritised, alongside consideration of admission avoidance pathways where appropriate. This may include greater utilisation of outpatient models such as same-day emergency care (SDEC) and coordinated community services to support safe management and reduce prolonged hospital admissions in similar cases.

• Finally, a further consideration is whether tocilizumab could be incorporated earlier within pathways, including as a first-line agent in complex patients and those deemed at high-risk of steroid-related complications. This approach would need to be balanced against current guideline recommendations, cost considerations, and the challenges of monitoring disease activity with IL-6 inhibition.

